# Bladder rupture: insights from a case series with a comprehensive literature review

**DOI:** 10.1093/jscr/rjaf341

**Published:** 2025-07-07

**Authors:** Hamza Ait Mahanna, Reda Safwat, Mehdi Safi-Eddine, Adil Kbiro, Amine Moataz, Mohamed Dakir, Adil Debbagh, Rachid Aboutaieb

**Affiliations:** Department of Urology, University Hospital Center Ibn Rochd, Casablanca, Morocco; Department of Urology, University Hospital Center Ibn Rochd, Casablanca, Morocco; Department of Urology, University Hospital Center Ibn Rochd, Casablanca, Morocco; Department of Urology, University Hospital Center Ibn Rochd, Casablanca, Morocco; Faculty of Medicine and Pharmacy of Casablanca, Casablanca, Morocco; Department of Urology, University Hospital Center Ibn Rochd, Casablanca, Morocco; Faculty of Medicine and Pharmacy of Casablanca, Casablanca, Morocco; Department of Urology, University Hospital Center Ibn Rochd, Casablanca, Morocco; Faculty of Medicine and Pharmacy of Casablanca, Casablanca, Morocco; Department of Urology, University Hospital Center Ibn Rochd, Casablanca, Morocco; Faculty of Medicine and Pharmacy of Casablanca, Casablanca, Morocco; Department of Urology, University Hospital Center Ibn Rochd, Casablanca, Morocco; Faculty of Medicine and Pharmacy of Casablanca, Casablanca, Morocco

**Keywords:** bladder rupture, emergency, urological trauma, case series

## Abstract

Bladder rupture, though infrequent, is a significant urological emergency with potential life-threatening implications. This report discusses seven cases, including three spontaneous and four traumatic ruptures. The spontaneous ruptures were associated with underlying conditions such as multiple sclerosis and Behçet’s disease, as well as risk factors like binge drinking. The traumatic ruptures resulted from blunt force injuries, including motor vehicle collisions and falls. Four cases exhibited intraperitoneal rupture, while three were extraperitoneal. Gross hematuria was consistently noted in traumatic cases, while spontaneous ruptures showed more varied presentations. All patients underwent CT cystography, highlighting its importance for diagnosis. Surgical intervention was successful for all, though one case required reoperation due to recurrence. This study emphasizes the need for timely imaging, tailored management strategies, and vigilance to improve outcomes in bladder rupture cases.

## Introduction

Bladder rupture is rare but can pose serious health risks, classified into spontaneous and traumatic types. Traumatic ruptures result from blunt or penetrating trauma, while spontaneous ruptures are usually linked to chronic distension or prior surgeries. Timely diagnosis is crucial to prevent complications [1].

Symptoms can vary, including lower abdominal pain, hematuria, urinary retention, or peritonitis, making diagnosis challenging. Imaging, particularly CT cystography, is key for accurate identification [2].

This article presents seven patients with bladder ruptures—two spontaneous and five traumatic. We aim to improve understanding and management strategies for this condition by analyzing their cases.

## Case series

This retrospective case series was carried out at the Urology Department of CHU Ibn Rochd throughout 2024. The study’s primary objective was to evaluate the clinical characteristics, diagnostic methodologies, and management outcomes of seven patients diagnosed with bladder rupture.

The study comprised seven consecutive patients diagnosed with bladder rupture during the designated study period. Among these cases, three were classified as spontaneous bladder ruptures, while the other four were of traumatic origin.

All patients involved gave informed consent, which secured approval to participate in the study and use anonymized data for research purposes.

Data were collected from the patients’ medical records, including:

- **Demographics**: Age and sex of the patients.
- **Clinical history**: Precipitating factors, comorbidities, and prior urological history.
- **Presentation**: Symptoms experienced and the time duration from onset to diagnosis.
- **Diagnostic evaluation**: Imaging results (including CT cystography and ultrasound) and relevant laboratory findings.
- **Rupture classification**: Classification of rupture types (spontaneous versus traumatic) and localization (intraperitoneal versus extraperitoneal).
- **Management**: An approach to treatment, whether surgical repair or conservative catheter drainage, was employed.
- **Outcomes**: Assessment of complications, length of hospital stay, and recovery status of patients.

Descriptive statistics were employed to summarize the data, emphasizing comparing clinical characteristics and outcomes between spontaneous and traumatic bladder rupture cases. Trends were analyzed, and results were displayed in the table below for enhanced clarity (see Table 1).

**Table 1:** Summary of patient demographics, rupture types, and clinical presentation

**Category**	**Parameter**	**Number**	**Percentage (%)**
Total cases	Total cases	7	100.0
Demographics	Mean age	40.1	
	Male	6	85.7
	Female	1	14.3
Rupture type	Spontaneous ruptures	3	42.9
	Traumatic ruptures	4	57.1
Intraperitoneal ruptures	Intraperitoneal ruptures (total)	4	57.1
	Intraperitoneal ruptures (spontaneous)	3	75.0
	Intraperitoneal ruptures (traumatic)	1	25.0
Extraperitoneal ruptures	Extraperitoneal ruptures (total)	3	42.9
	Extraperitoneal ruptures (traumatic)	3	75.0
Clinical presentation	Acute lower abdominal pain	7	100.0
	Oliguria	2	28.6
	Hematuria (total)	5	71.4
	Hematuria (spontaneous)	1	33.3
	Hematuria (traumatic)	4	100.0

### Patient demographics and clinical characteristics

*Spontaneous Bladder Rupture:* In a cohort of three patients diagnosed with spontaneous bladder rupture, all presented with acute lower abdominal pain, resulting in a prevalence of 100%. Hematuria and oliguria were observed in 50% of the cases. Noteworthy contributing factors included:

1. A diagnosis of multiple sclerosis in one patient.
2. Behçet’s disease in another patient.
3. A history of binge drinking in the third case.

These observations highlight the potential involvement of underlying neurological, autoimmune, and acute lifestyle-related factors in the pathogenesis of spontaneous bladder rupture.

*Traumatic Bladder Rupture:* In a cohort of four patients diagnosed with traumatic bladder rupture, all incidents were attributable to blunt trauma, resulting in a prevalence rate of 100%. The mechanisms of injury were categorized as follows:

1. Motor vehicle collisions in two patients (50%).
2. Falls from a height in two patients (50%).

### Clinical presentation

Spontaneous Bladder Rupture: Three patients had acute lower abdominal pain (100%). Hematuria and oliguria occurred in 50%. Contributing factors included chronic bladder distension (66.7%) and binge drinking (33.3%).

### Diagnostic findings

All seven patients underwent CT cystography as the primary imaging modality, which revealed contrast extravasation in all cases (100%), thereby confirming the diagnosis of bladder rupture. However, preliminary imaging was performed in some cases: Ultrasound (71.4%) helped assess free fluid, suggesting hemoperitoneum in two cases. X-ray (42.9%) was used when pelvic fractures were suspected but was inconclusive for bladder rupture and urinalysis revealed gross hematuria in two cases, prompting further imaging.

**Figure 1:**
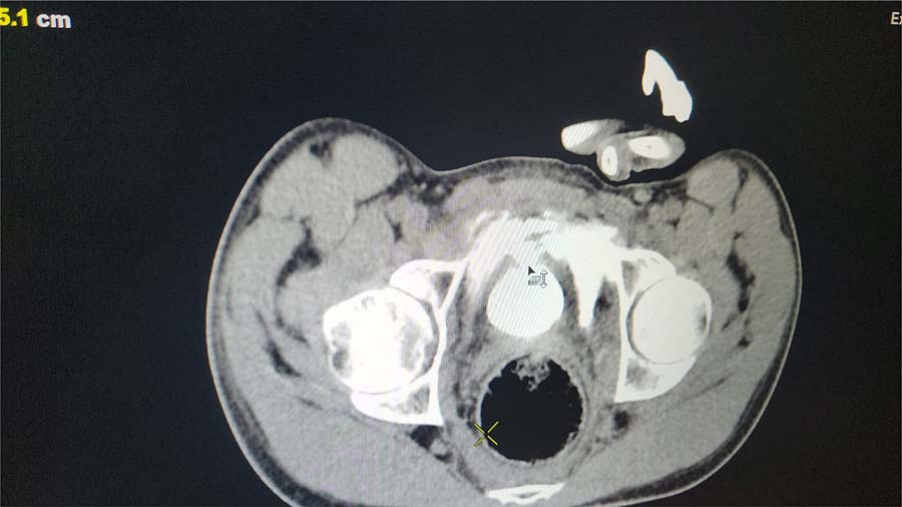
CT scan reveals contrast material leaking from the bladder lumen into the surrounding pelvic cavity, indicating a bladder rupture.

Out of the total cases, four intraperitoneal ruptures (57.1%) and three extraperitoneal ruptures (42.9%) were observed. This highlights the significance of CT cystography in differentiating between these rupture types to inform management strategies (see Fig. 1).

CT cystography proved highly effective for timely and accurate diagnoses, with no reported misdiagnoses or delays. This imaging technique is the gold standard for identifying and classifying bladder ruptures as intraperitoneal or extraperitoneal.

### Management

**Figure 2:**
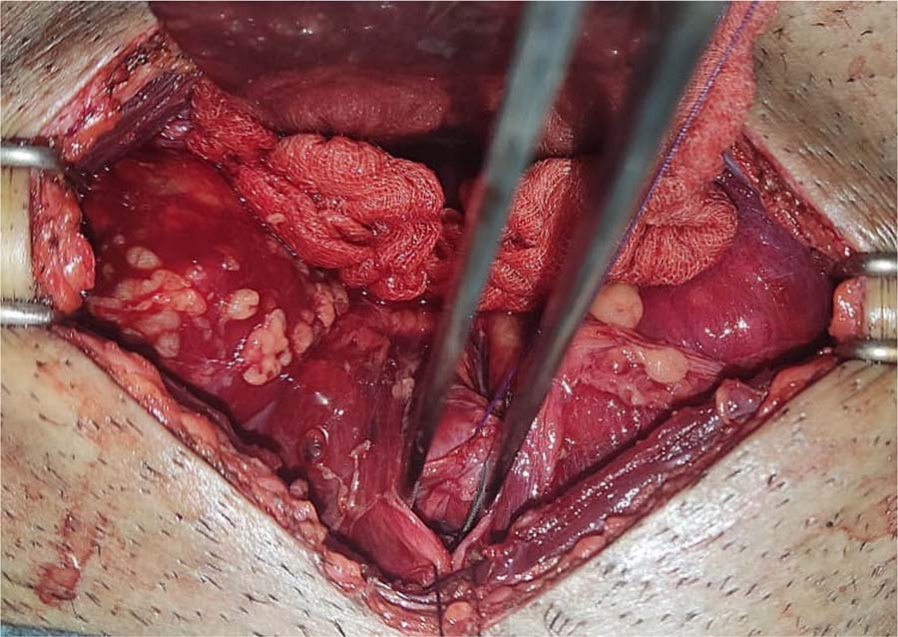
The image shows an extraperitoneal bladder rupture, revealing a tear in the bladder wall and damage to surrounding tissues.

**Figure 3:**
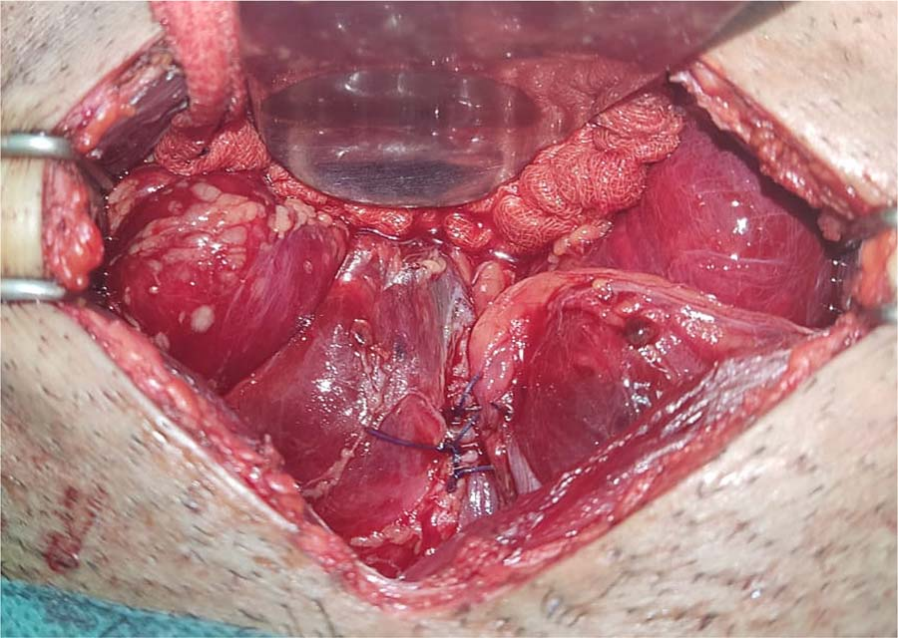
The image depicts the bladder after surgical repair, showing the successfully sutured rupture site and restored continuity.

All seven cases of bladder rupture both spontaneous and traumatic were treated surgically via exploratory laparotomy to repair the injury (see Fig. 2). In both types of cases, the ruptured bladder was successfully closed (see Fig. 3). Postoperatively, Redon drain was removed on the second day, and a urinary catheter was maintained for 3 weeks to aid healing.

Most patients experienced uncomplicated recoveries, but one patient (14.3%) experienced a recurrence of bladder rupture, requiring further intervention. This recurrence was managed by a secondary surgical repair via exploratory laparotomy, followed by prolonged catheterization for 4 weeks to ensure adequate bladder healing. Postoperative follow-up showed successful recovery with no further complications.

## Discussion

Bladder rupture is an uncommon urological emergency, and spontaneous occurrences make up an even smaller percentage of bladder ruptures, comprising less than 1% [3].

### Spontaneous bladder ruptures

Spontaneous rupture of the urinary bladder (SRUB) is a rare but serious condition that can lead to complications if not promptly diagnosed and managed. It occurs without external trauma and is often associated with chronic bladder distension, neurogenic bladder dysfunction, and heavy alcohol use [4]. SRUB presents diagnostic challenges due to nonspecific symptoms like lower abdominal pain, urinary retention, and hematuria, which can mimic other acute abdominal issues [5].

Our study’s clinical and demographic characteristics of SRUB cases align with existing literature, revealing key risk factors and presentations. We identified three predisposing conditions: neurogenic bladder from multiple sclerosis, Behçet's disease, and risk factors such as acute alcohol intoxication. These findings support previous reviews that highlight chronic bladder dysfunction and systemic inflammatory diseases as significant contributors [4, 6]. Among these, acute alcohol intoxication stands out as a major factor linked to spontaneous bladder rupture, accounting for 11%–39% of documented cases [7–9].

The mechanisms underlying this risk are multifaceted. Alcohol acts as a diuretic, causing rapid bladder filling that may exceed normal limits while also impairing both the central and peripheral nervous systems’ ability to recognize cues for urination [10]. Prolonged overdistension of the bladder, especially at the weaker dome, increases the risk of rupture. Alcohol consumption can further heighten this risk by diminishing attention to physiological signals during heavy drinking [7].

The dome of the bladder is particularly prone to rupture due to its thinner wall and vulnerability to increased pressure from overdistension [11]. Our analysis found that all spontaneous bladder rupture cases resulted in intraperitoneal ruptures, accounting for approximately 89% of all cases [11, 12].

Spontaneous bladder rupture presents with nonspecific signs and symptoms, making diagnosis challenging. The most common symptom is acute abdominal pain, reported in about 83% of cases [8, 12]. Hematuria, seen in half of our spontaneous cases, is an essential diagnostic indicator but is not always present; large-scale reviews indicate it occurs in about 50%–60% of cases. Other symptoms, such as oliguria, anuria, or urinary retention, may occur, but they are not definitive [9].

When cases involve alcohol intoxication, patients may present with vague symptoms, leading to a delay in recognizing the bladder rupture and altered mental status [8]. Nonspecific symptoms like nausea, vomiting, and abdominal discomfort can lead to misdiagnoses related to gastrointestinal issues or acute kidney injury [7]. Therefore, maintaining a high level of clinical suspicion and utilizing prompt imaging techniques are essential for diagnosing SRUB in such cases [8, 9].

### Blunt trauma

Blunt force trauma is the primary cause of bladder ruptures, with motor vehicle accidents being the most common trigger [13, 14]. This type of injury frequently occurs when the bladder is full, making it especially susceptible to increased pressure and compression between the abdominal wall and the pubic symphysis [13, 14]. In our study, every instance of traumatic bladder rupture was attributed to blunt force trauma, which contrasts with previous studies.

Although our study found a nearly equal distribution of intra- and extraperitoneal ruptures, previous studies indicate that extraperitoneal ruptures are generally more common in traumatic cases. Typically occurring at the bladder’s dome, intraperitoneal ruptures are linked to high-energy trauma, particularly in individuals with a distended bladder at the moment of injury. These types of ruptures pose significant risks, including peritonitis, chemical ileus, and sepsis, due to urine leaking into the peritoneal cavity [13–15]. They are often caused by compressive forces during vehicular accidents, as highlighted in our analysis and broader reviews [15, 16].

In contrast, extraperitoneal ruptures are usually linked to pelvic fractures. They are frequently the result of shear forces that disrupt the bladder at its fixed points or due to direct injury from bony fragments. These types of ruptures generally affect the bladder’s base and anterior wall and are less likely to lead to systemic complications when treated correctly [13–15]. Current guidelines recommend conservative management for isolated extraperitoneal ruptures in stable patients, with catheter drainage being an effective method for facilitating healing in most instances.

Diagnosing bladder rupture can be challenging due to its nonspecific clinical presentation, whether it occurs spontaneously or due to trauma. In cases of spontaneous rupture, patients typically experience sudden abdominal pain and may not exhibit hematuria, which makes early diagnosis more complicated [11, 17]. In instances of blunt trauma, the presence of gross hematuria is a significant sign, although it might be absent in up to 44% of cases [15].

Historically, retrograde cystography was the standard diagnostic tool for identifying traumatic bladder ruptures. However, CT cystography has become preferred because it offers similar specificity while providing additional benefits [18]. It delivers high diagnostic accuracy and can differentiate between intraperitoneal and extraperitoneal ruptures, which is crucial for determining the appropriate management approach [13, 14]. Unlike traditional cystography, CT is less invasive, more straightforward to perform, and can also identify other abdominal issues while ruling out bladder rupture [19]. Additionally, emerging technologies such as machine learning hold potential for improving diagnostic efficiency, though they are still in the experimental phase [15].

Surgical repair was performed in all cases regardless of the underlying cause, resulting in positive outcomes. This aligns with the general agreement that intraperitoneal ruptures necessitate surgical intervention to prevent peritonitis, while conservative treatment can be adequate in certain extraperitoneal situations [4, 6, 12]. The recurrence of rupture in one patient highlights the importance of diligent postoperative monitoring, as studies indicate that recurrence rates tend to be higher with conservative management compared to surgical methods [11].

In our study, the recovery process after surgery was predominantly smooth. Drains were removed by the second postoperative day, and catheters remained in place for 3 weeks. This standardized approach likely contributed to the absence of significant complications, and it is supported by other studies that stress the significance of careful postoperative care [6, 12].

This series of cases highlights the necessity of maintaining a keen awareness of potential bladder rupture, especially in individuals with risk factors like neurological conditions, autoimmune disorders, or prior blunt trauma. The significance of CT cystography for prompt and precise diagnosis is paramount, as it plays a crucial role in shaping management strategies and influencing patient outcomes.

Bladder rupture presents a significant challenge both in diagnosis and treatment, necessitating immediate imaging and intervention. This collection of cases emphasizes the effectiveness of surgical treatment while underlining the need for tailored approaches based on the cause and nature of the rupture. Additional research involving larger participant groups and extended follow-up periods is essential to enhance management techniques and overall patient outcomes.

## References

[1] Simon LV, Sajjad H, Lopez RA, Burns B. Bladder rupture. In: StatPearls [Internet]. Treasure Island (FL): StatPearls Publishing; 2024 http://www.ncbi.nlm.nih.gov/books/NBK470226/29262195

[2] Su PH, Hou SK, How CK, et al. Diagnosis of spontaneous urinary bladder rupture in the ED. Am J Emerg Med 2012;30:379–82. 10.1016/j.ajem.2011.10.003 22204994

[3] Al-Nahawi AA, Alsuwailim AM, Alhassawi AS, et al. Spontaneous rupture of the urinary bladder in an elderly diabetic male. Cureus 2023;15:e46481. 10.7759/cureus.46481 37927748 PMC10624327

[4] Zhao S, Duan H, Wang Y, et al. Spontaneous rupture of the urinary bladder: a rare case report. Heliyon 2023;9:e17129. 10.1016/j.heliyon.2023.e17129 37455977 PMC10338306

[5] Kunichika H, Takahama J, Taguchi H, et al. The diagnostic challenge of non-traumatic bladder rupture: a pictorial essay. Jpn J Radiol 2023;41:703–11. 10.1007/s11604-023-01395-1 36729190 PMC10313837

[6] Kurniawan D, Pramod SV. Spontaneous intraperitoneal bladder rupture managed conservatively. Urol Case Rep 2022;45:102220. 10.1016/j.eucr.2022.102220 36164382 PMC9508409

[7] Merga OT, Bayileyegn NS. Spontaneous bladder rupture after alcohol binge presenting as a rare cause of acute abdomen: a case report and review of literatures. Int J Surg Case Rep 2023;111:108942. 10.1016/j.ijscr.2023.108942 37820482 PMC10570937

[8] Devkota S, Adhikari S, Singh M, et al. Spontaneous atraumatic rupture of the urinary bladder following alcohol intoxication: a rare case report. Clin Case Rep 2024;12:e9395. 10.1002/ccr3.9395 39219775 PMC11362228

[9] Miller J, McCague A. Spontaneous bladder rupture after binge drinking. Cureus 2025;15:e48107. 10.1097/JTN.0000000000000854 PMC10690061 38046710

[10] Herd AM, Crofts NG, Lee LM, et al. Isolated bladder rupture after minor trauma in a patient with alcohol intoxication. J Emerg Med 1994;12:409–11. 10.1016/0736-4679(94)90287-9 8040601

[11] Reddy D, Laher AE, Lawrentschuk N, et al. Spontaneous (idiopathic) rupture of the urinary bladder: a systematic review of case series and reports. BJU Int 2023;131:660–74. 10.1111/bju.15974 36683400

[12] Zhang Y, Yuan S, Alshayyah RWA, et al. Spontaneous rupture of urinary bladder: two case reports and review of literature. Front Surg 2021;8:721705. 10.3389/fsurg.2021.721705 34796196 PMC8592997

[13] Elkbuli A, Ehrhardt JD, Hai S, et al. Management of blunt intraperitoneal bladder rupture: case report and literature review. Int J Surg Case Rep 2019;55:160–3. 10.1016/j.ijscr.2019.01.038 30739872 PMC6369329

[14] Mahat Y, Leong JY, Chung PH. A contemporary review of adult bladder trauma. J Inj Violence Res 2019;11:101–6. 10.5249/jivr.v11i2.1069 30979861 PMC6646823

[15] Hertz AM, Hertz NM, Johnsen NV. Identifying bladder rupture following traumatic pelvic fracture: a machine learning approach. Injury 2020;51:334–9. 10.1016/j.injury.2019.12.009 31866131

[16] Trang VAV, Le NHD, Nguyen DTS, et al. Delayed bladder rupture following blunt trauma: a case report and literature review. Trauma Case Rep 2024;54:101110. 10.1016/j.tcr.2024.101110 39318765 PMC11420461

[17] Sholklapper T, Elmahdy S. Delayed diagnosis of atraumatic urinary bladder rupture. Urol Case Rep 2021;38:101723. 10.1016/j.eucr.2021.101723 34094877 PMC8167143

[18] Quagliano PV, Delair SM, Malhotra AK. Diagnosis of blunt bladder injury: a prospective comparative study of computed tomography cystography and conventional retrograde cystography. J Trauma Acute Care Surg 2006;61:410–22. 10.1097/01.ta.0000229940.36556.bf 16917459

[19] Muneer M, Abdelrahman H, El-Menyar A, et al. Spontaneous atraumatic urinary bladder rupture secondary to alcohol intoxication: a case report and review of literature. Am J Case Rep 2015;16:778–81. 10.12659/AJCR.894992 26522816 PMC4634162

